# Unraveling the Potential of Organic Oregano and Tarragon Essential Oils: Profiling Composition, FT-IR and Bioactivities

**DOI:** 10.3390/plants12234017

**Published:** 2023-11-29

**Authors:** Dan Vârban, Marius Zăhan, Ioana Crișan, Carmen Rodica Pop, Emese Gál, Răzvan Ștefan, Ancuța Mihaela Rotar, Adriana Sebastiana Muscă, Ștefania Dana Meseșan, Vasile Horga, Ioan Ladoși, Loredana Olar, Andrei Stoie, Rodica Vârban

**Affiliations:** 1 Department of Crop Science, Faculty of Agriculture, University of Agricultural Sciences and Veterinary Medicine of Cluj-Napoca, Calea Mănăștur Street No. 3–5, 400372 Cluj-Napoca, Romania; dan.varban@usamvcluj.ro (D.V.); ioana.crisan@usamvcluj.ro (I.C.); vasile.horga@usamvcluj.ro (V.H.); andrei.stoie@usamvcluj.ro (A.S.); 2 Department of Biotechnology, Faculty of Animal Science and Biotechnology, University of Agricultural Sciences and Veterinary Medicine of Cluj-Napoca, Calea Mănăștur Street No. 3–5, 400372 Cluj-Napoca, Romania; mzahan@usamvcluj.ro (M.Z.); adriana-sebastiana.musca@usamvcluj.ro (A.S.M.); stefania.mesesan@usamvcluj.ro (Ș.D.M.); ioan.ladosi@usamvcluj.ro (I.L.); 3 Biotechnology Research Center, Life Sciences Institute “King Michael I of Romania”, University of Agricultural Sciences and Veterinary Medicine of Cluj-Napoca, Calea Mănăștur Street No. 3–5, 400372 Cluj-Napoca, Romania; 4 Department of Food Science, Faculty of Food Science and Technology, University of Agricultural Sciences and Veterinary Medicine of Cluj-Napoca, Calea Florești No. 64, 400509 Cluj-Napoca, Romania; carmen-rodica.pop@usamvcluj.ro (C.R.P.); anca.rotar@usamvcluj.ro (A.M.R.); 5 Department of Chemistry and Chemical Engineering, Hungarian Line, Faculty of Chemistry and Chemical Engineering, Babeș-Bolyai University, Arany János No. 11, 400028 Cluj-Napoca, Romania; emese.gal@ubbcluj.ro; 6 Department of Preclinical Sciences, Faculty of Veterinary Medicine, University of Agricultural Sciences and Veterinary Medicine of Cluj-Napoca, Calea Mănăștur Street No. 3–5, 400372 Cluj-Napoca, Romania; rstefan@usamvcluj.ro (R.Ș.); loredana.olar@usamvcluj.ro (L.O.)

**Keywords:** aromatic, antibacterial, cytotoxic, volatiles, health, food, medicinal

## Abstract

Oregano and tarragon are widely cultivated culinary herbs used for food seasoning, having familiar characteristic aromas appreciated by the wide public. The aim of this research was to characterize essential oils (EOs) from locally sourced organic oregano and tarragon (Cluj, Romania) and study their bioactivity potential. Results showed that oregano EO had a sesquiterpene dominant profile responsible for strong bands between 2800 and 3000 cm^−1^ on the Fourier transform infrared spectroscopy (FT-IR) spectrum and a composition consistent with reports from similar climatic regions. The tarragon EO profile was defined by phenylpropanoids responsible for the strong sharp peaks between 1000 and 1600 cm^−1^ on the FT-IR spectrum. In oregano EO, 22 compounds were identified with β-caryophyllene as a major constituent. In tarragon EO, 20 compounds were identified with eugenol as a major constituent. Oregano EO had a stronger antibacterial effect against both Gram-negative and Gram-positive bacterial strains, while tarragon EO had a slightly stronger cytotoxic effect on three types of cancer cell lines tested (skin melanoma, prostate carcinoma, and colorectal adenocarcinoma). It was concluded that, given the fact that a sufficient supply of high-quality plant material can be available for EO extraction, culinary herbs can become reliable candidates for many industries without the risk of discontinued supply. Therefore, research aiming to widen their potential applications is welcome and worth pursuing.

## 1. Introduction

Plants have always been a source of well-being and health-promoting products. For a wide range of needs, humans have turned to plants. Among the vast array of aromatic plant species known, culinary herbs and spices have been most appreciated and valued throughout time, foremost as food and medicines, which remain important drivers of market demand, alongside many other secondary or novel applications [1]. Essential oils (EOs) are complex mixtures containing volatile organic compounds of low molecular weight belonging to various chemical classes, with the vast majority being from the terpene family. These are produced and localized within the cytoplasm of certain plant differentiated structures, mainly trichomes. These structures vary by species in their morphology and mechanism of synthesis release, while their density varies according to plant organ [2]. Depending on their preferences, people find the aroma of various EOs pleasing, comforting or relaxing, and ancient evidence for the extraction and uses of EOs from plants indicates that, since early times, people have appreciated them for practical and spiritual reasons. Today, about 40,000–60,000 tons of EOs are produced annually, for which the prices are mainly influenced by plant species, quality, destination of use and extraction methods employed [3]. Consumers are more health-conscious and have increasing quality expectations, which puts pressure on market trends. In this context, EOs obtained from plants, including culinary herbs, have risen in importance and the areas of applications are expanding. Particularly, EOs have become the preferred natural replacers for compounds of synthetic origin in many food and non-food products [4].

EOs have therapeutic activities, including anti-inflammatory, immunomodulant, and antimicrobial to name a few [5]. Cosmetic, pharmaceutical [6], and fragrance [7] industries remain highly interested in applications of EOs. The role of EOs in food flavoring and animal nutrition are also increasing [8]. For example, a growing interest in these products is manifested by pig producers due to beneficial effects on piglet survival in their early life [9]. EOs can be used as natural additives for food packaging as films or coating, preventing spoiling and prolonging shelf life [10].

Culinary herbs are the best candidates for EO production for several reasons. (1) Their cultivation technology is already well established and can ensure sufficient quantities of plant material without difficulty. (2) Because they are widely cultivated and are not difficult to procure locally, the risk of supply shortage is reduced, thus not causing discontinued availability but, on the contrary, covering sustained or increased demand. (3) People are familiar with their aroma since they are commonly used for seasoning dishes and meals, therefore, being pleasant or acceptable for the wider public not just niche consumers. Any of these reasons make this category of plant species particularly attractive for various industries interested in providing applications based on EOs from food to medicine and agriculture.

*Origanum vulgare* L., commonly called oregano, is a culinary herb from the family Lamiaceae. It is a perennial plant native to the Mediterranean region, with several subspecies found in various regions of Europe and Western Asia. The crop can ensure two to three harvests in a vegetative season. Between the stages of 50% flowering and full bloom, plants can be harvested for EO production [11]. Plants are covered with microscopic peltate and capitate trichomes, where the EO is accumulated [12]. The EO concentration ranges from 1.1 to up to 8.2% [13].

*Artemisia dracunculus* L., commonly called tarragon, is a culinary herb from the family Asteraceae. It is a perennial plant native to Siberia and Mongolia but is widespread in cultivation. The crop can ensure two to three harvests in a vegetative season. At the leaf budding stage and the beginning of flowering, the plants have the highest concentration of EO [14]. Leaves, stems, and inflorescence present biseriate glandular trichomes [15]. The EO concentration ranges between 0.15 and 3.1% [16].

The aim of the research was to provide a comparative characterization of EOs from two culinary herbs belonging to different botanic families, sourced locally, and screen their biologic potential.

To reach this aim, four objectives were defined:

- Describing the volatile profile of the two EOs;
- Identifying distinctive FT-IR bands;
- Determining the antibacterial activity;
- Screening cytotoxicity on cell lines.

## 2. Results

### 2.1. GC-MS Volatile Profile

For the oregano EO, 22 compounds were identified. The major volatile compound was β-caryophyllene, a natural sesquiterpene. Spathulenol, 4-terpineol, sabinene, and β-cubebene also were found in relatively large quantities, which characterizes this type of essential oil. Compounds like β-bourbonene, γ-muurolene, and γ-elemene from the sesquiterpenes were also present (Table 1).

In the tarragon EO, 20 volatile compounds were identified, predominantly methyl eugenol, elemicin, and isoelemicin as major compounds, classified as phenylpropene, a type of phenylpropanoid. Furthermore, components from the bicyclic monoterpenes were found as well in the analyzed EO. Among them, sabinene and monoterpenoids like terpinene-4-ol, α-terpineol, carvone, and β-citronellol were present as minor compounds. Monoterpenes such as ocimene isomers and geranyl acetate were also found (Table 1).

### 2.2. FT-IR Spectra

The two EO profiles were defined by a different major class of constituents (accounting for >50% of EO composition): oregano EO had a sesquiterpene defined profile, while the tarragon EO profile was defined by phenylpropanoids. Common to both EOs was the comparable monoterpene/monoterpenoids content, with sabinene contribution exceeding 5% but being less than 10% in both cases (Table 1).

**Figure 1:**
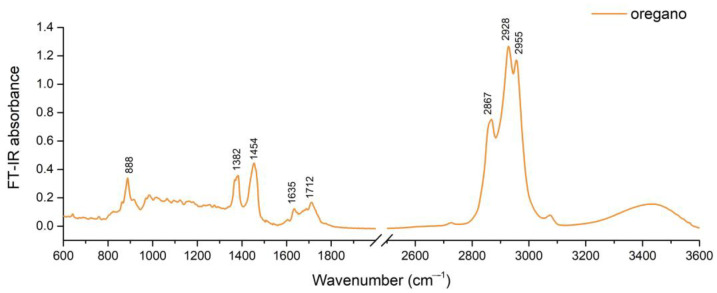
FT-IR spectra of oregano EO.

The constituents of oregano EO were β-caryophyllene with a concentration exceeding 20%, followed by sabinene with just above 6% (Table 1). They are expected to be imprinting a major influence on the FT-IR spectrum compared to lower concentration constituents (Figure 1). It can be observed that oregano EO presents the strongest FT-IR absorption between 2800 and 3000 cm^−1^, where a strong band having three noticeable peaks (Figure 1) is present. These could be assigned to C–H stretch from the molecule of β-caryophyllene, superimposed by sabinene with contribution particularly to the 2867 cm^−1^ and, respectively, β-cubebene contribution to 2955 cm^−1^.

The peak at 888 cm^−1^ from the oregano FT-IR spectrum can be attributed to C–H out-of-plane bending from the structure of β-caryophyllene [17]. The peak at 1382 cm^−1^ can be attributed, however, to CH_3_ symmetrical deformation and it was found to be distinguishing oregano EO from some members of the Lamiaceae botanical family, while the peak at 1454 cm^−1^ can be attributed to CH_3_ asymmetrical deformation [18]. The lower intensity peaks at 1636 cm^−1^ and 1712 cm^−1^ could be attributed to C=C stretch and, respectively, to C=O stretch, as displayed by EOs from other widely cultivated species of this botanic family [19].

**Figure 2:**
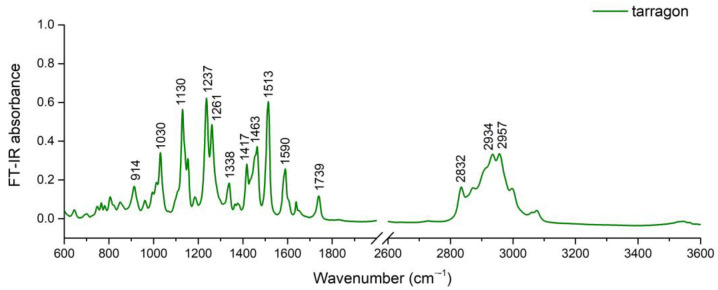
FT-IR spectra of tarragon EO.

Tarragon EO presented three major phenylpropanoid compounds, together exceeding 50% of volatile profile: methyl eugenol, elemicin, and iso-elemicin (Table 1). Therefore, it is inferred that these functional groups imprinted a major influence on the spectrum compared to the minor constituents and caused the sharp peaks between 1000 and 1600 cm^−1^. A broad band was found between 2800 and 3100 cm^−1^ (Figure 2).

Based on assignments for FT-IR spectra of *Artemisia* species EOs [20,21,22], main peaks identified in tarragon EO FT-IR spectrum were assigned to functional groups as follows. The peak at 914 cm^−1^ was assigned to out-of-plane vibration of =C–H in aromatics [20] and the peaks between 1030 and 1154 cm^−1^ to C–O or C–O–C stretching, respectively [20,22]. The peak at 1237 cm^−1^ was assigned to stretching of molecular bonds from C–O–C of aromatic acid ester as well as to phenolic C–OH group vibration [20]. Peaks at 1417 and 1463 cm^−1^ could be assigned to C–H asymmetric and symmetric bending or O–H bend from phenol and tertiary alcohol. The peak at 1739 cm^−1^ can be attributed to C=O stretch of bonded conjugated ketones, aldehydes, and esters [20]. The peaks from region 2830 to 2960 cm^−1^ were assigned to –CH vibration modes in SP^2^ and SP^3^, as described for tarragon EO [21].

### 2.3. Antibacterial Potential

The results showed that oregano EO had a stronger antibacterial effect than tarragon EO against the tested strains, with a statistically significant difference for all tested assays (Table 2). Because the tested bacteria are common microbiological contaminants of food products, these results hint to potential applications for the food industry.

The EO of oregano gives the lowest MICs against *L. monocytogenes*, followed by *S. aureus* and evenly by *E. coli*. Gram-positive bacteria are more resistant to tarragon and oregano EOs. However, tarragon EO manifested similar behavior against both Gram-positive (*S. aureus*) and Gram-negative (*E. coli*) bacteria.

### 2.4. Cytotoxic Effect on Cell Lines

Microscopic analysis of cell morphology revealed a large number of detached and shrunken cells after exposure to higher concentrations of EOs. This process was in general more evident for the concentrations of 0.032% and 0.064% of both oregano and tarragon EOs in all cancer cell lines.

**Figure 3:**
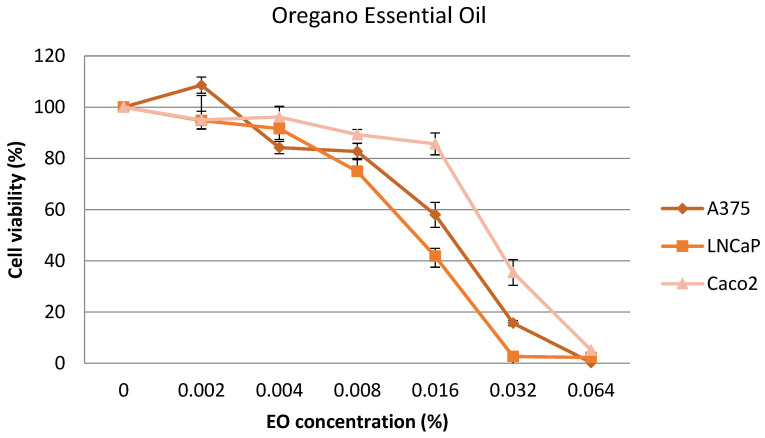
Cytotoxicity of A375, LNCaP, and Caco2 cell lines treated with oregano EO (0.002–0.064%) for 24 h.

The cytotoxic effect of the oregano and tarragon EOs was evaluated by performing the 3-(4,5-dimethyl-2-thiazolyl)-2,5-diphenyl-2H-terazolium bromide (MTT) assay, which detects dehydrogenase activity in viable cells. The results were expressed as percentage of the control. Oregano EO cytotoxicity on A375, LNCaP, and Caco2 cell lines is shown in Figure 3. The IC_50_ values (median inhibitory concentration that causes ~50% cell death) varied between 0.023% in the case of prostate carcinoma and 0.032% in the case of colorectal adenocarcinoma. The IC_50_ value for skin melanoma was 0.026%.

**Figure 4:**
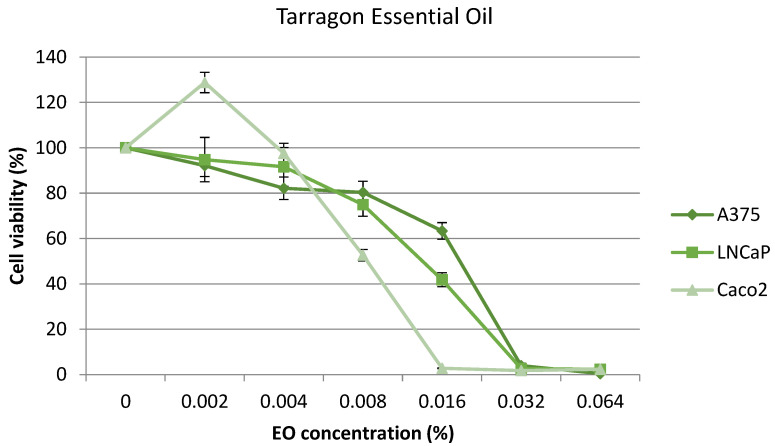
Cytotoxicity of A375, LNCaP, and Caco2 cell lines treated with tarragon EO (0.002–0.064%) for 24 h.

Cytotoxic activities of tarragon EOs on the Caco2, A375, and LNCaP cell lines are shown in Figure 4. More similar cytotoxicity values of concentration were found to induce IC_50_ in the case of the tarragon EO than in the oregano EO, independent of cell line. The IC_50_ values were 0.021% in the case of colorectal adenocarcinoma, 0.023% for prostate carcinoma, and 0.024% for skin melanoma.

It could be observed that, for LNCaP cell line, the IC_50_ value was the same (0.023%) for both oregano and tarragon EOs, while, for Caco2, the IC_50_ value was the highest for oregano EO (0.032%), respectively, and the lowest for tarragon EO (0.021%). Furthermore, it seems that tarragon EO is slightly more cytotoxic than oregano EO for the tested cancer cell lines (0.023 vs. 0.027%).

## 3. Discussion

This research presents results of EOs analyses from two common culinary herbs: oregano and tarragon, which belong to different botanic families that can explain the difference between them in terms of composition and FT-IR spectra. The EOs were obtained from organic crops sourced locally; therefore, all stages from cultivation to extraction conditions and testing were documented. No inputs were applied to the crop (no fertilizer and no chemicals) in order to reduce any potential influence on the quality of EOs. We suppose this increases the reliability of the findings, the chemical composition being insightful for the quality of EOs that can be achieved in the pedo-climatic conditions of this study.

Chemical and FT-IR profiling were complementary for highlighting the differences between the two EOs regarding the main classes of compounds. Oregano EO had a sesquiterpene-defined profile, while the tarragon EO profile was defined by phenylpropanoids.

FT-IR investigation of 30 EOs from different botanic families, including Lamiaceae and Asteraceae, showed that terpenoid components found in EOs produce characteristic bands at ~1100 cm^−1^ due to C–O stretch, ~1700 cm^−1^ due to C=O stretch, ~2900 cm^−1^ due to C–H stretch, and broad band ~3400 cm^−1^ due to O–H stretch, with distinct clusters consistent with botanic families [18], indicating the discriminant power of this method.

The EO components identified in the samples belong to two main biosynthetic groups: terpenes (including monoterpenes, sesquiterpenes, and derivatives) and phenylpropanoids, which are compounds displaying an aromatic ring with a propene tail [23]. These have different biosynthesis pathways: while terpenes derive from the methylerythritol phosphate (MEP) and mevalonic acid (MVA) pathways, by comparison, the shikimic acid biosynthetic pathway generates phenylpropanoids [24]. Furthermore, the location of the two main terpene synthesis pathways in plants differs as well: MVA path is active in cytosol, giving rise to sesquiterpenes and triterpenes, while MEP path is active in plastids, giving rise to monoterpenes, diterpenes, and tetraterpenes. The shikimate path occurs in plastids but produces a wider range of compound classes, not limited to EO components. The volatile phenylpropanoids molecules do not display units as terpenes do and have a wide range of effects, including psychoactive [23].

Some phenylpropanoids are accepted food flavorings; others have been prohibited as food flavoring agents due to evidence on their hepatotoxicity yet still naturally occur in some spices [23,25]. In this study, only tarragon EO presented volatile phenylpropanoids, which were not present in oregano EO. Noticeably, tarragon EO presented high amounts of methyl eugenol (Table 1), a compound reported so far in over 40 species from Asteraceae family (including several *Artemisia* species). In the life of the plant, this compound has a role in defense against herbivores and some insects because it has a deterrent effect when released by the damaged plant [26]. Methyl eugenol occurrence and concentration in some *Artemisia* species can depend on phenophase; yet, in *A. dracunculus*, the highest content was identified at mass flowering [27], the phenophase in which *herba* was collected for EO extraction in this study. Elemicin (another phenylpropanoid compound which has psychoactive effects) was present in tarragon EO (Table 1). This compound has not shown fluctuations across growth stages of tarragon [27]. Elemicin has wide pharmacological activities such as antimicrobial, antioxidant, and antiviral effect and is found in other culinary plants as well, such as nutmeg (*Myristica fragrans*) or parsley (*Petroselinum crispum*) to name the most common ones. A study on mice showed that metabolic activation plays an important role in elemicin-induced cytotoxicity [28].

Oregano from Mediterranean countries are known for their high-quality EO. Specifically, Greek oregano (*O. vulgare* subsp. *hirtum*), a warm-climate plant, accumulates mainly phenolic monoterpenes (thymol and carvacrol) [29]. According to Kokkini and collaborators [30], Greek oregano can be divided into the following groups: linalool, terpinen-4-ol, and sabinene hydrate chemotype; carvacrol and/or thymol chemotype; and the sesquiterpene chemotype, respectively. The common oregano, which is much more widespread across Europe (*O. vulgare* subsp. *vulgare* as well as other subspecies), were shown to display extremely high variation in EO composition. This was found following a screening of oregano populations Europe-wide. Several distinctive oregano chemotypes were defined: cymyl chemotype, sabinyl chemotype, acyclic chemotype, and some mixed chemotypes of the different pathways named above and consistent with a geographical gradient. Particularly, sabinyl chemotype was frequent in countries of Central, Northern, and Eastern Europe and had a high proportion of sesquiterpenes [31], as found in this this study as well. Furthermore, in the same study, Lukas and collaborators (2015) showed that oregano populations in Europe had β-caryophyllene in higher amounts out of the sesquiterpenes, which is consistent with the findings reported here. A study in conditions from Russia (South Siberia) indicated that, in oregano EO (*O. vulgare* subsp. *vulgare*), the oxygenated sesquiterpene-caryophyllene oxide defined the volatile profile of all oregano samples, regardless of plant organ or shade conditions. In addition, sabinene was among the next major compounds with some variations with organ and treatment [32]. In this study conducted in conditions of central Romania, having also a humid continental climate like the one from the study cited above, β-caryophyllene and sabinene were the major compounds of oregano EO (Table 1). Therefore, the overall quality of EO from this study is somewhat similar to plants grown in regions with comparable climatic conditions.

Oregano EO composition variation could be associated with the genotypic diversity. In a recent study carried out by Jianu and collaborators [33], on the EO of *O. vulgare* var. *aureum* grown in conditions of Hunedoara county, situated also in the Transylvanian historical region of Romania, a chemical composition different from the one we found was reported. They reported the major EO compounds being represented by γ-terpinene, para-cymene, germacrene, β-trans-ocimene, and cis-β-ocimene. Morshedloo and collaborators [34] found four chemotypes across seven populations of *O. vulgare* cultivated in the same agricultural conditions in Iran, as further evidence for the wide variations in EO within this species. Oregano EOs obtained from other species—*O. dictamnus*, *O. microphyllum*, *O. libanoticum* [35], and *O. glandulosum* [36]—showed promising biological activities but also differences in their chemical composition.

The large variation in the composition of oregano EOs is posing some challenges in interpreting their biologic activities, since their composition can vary substantially. Therefore, the observed effects could be due to different compounds. Thus, perhaps the best comparison approach is to associate the effect reported with the major compounds and classes of compounds identified in the tested EO sample in each case.

Among the oregano EO constituents, carvacrol and/or thymol were often associated with antibacterial effect, although their proportion was shown to vary extremely from 0% to over 80% of EO composition for either of them, depending on chemotype, as reported by studies in various countries [37]. These two compounds were absent in oregano chemotype from this study. However, β-caryophyllene, the major compound identified, was shown to possess a wide biologic activity, including antibacterial as well as beneficial effects in the case of numerous neurodegenerative and inflammatory pathologies [38]. Furthermore, we noticed that the antimicrobial effect registered here for oregano EO was better than the ones we demonstrated in three other species from this botanic family (Lamiaceae) such as lavender, sage, and basil cultivated in similar conditions [19].

The biological activity of EOs including antimicrobial, antioxidant, anti-inflammatory, antiproliferative, antitumor, antimutagenic, sedative, diuretic, and regenerating activity was investigated in recent years [39,40,41,42,43,44,45,46]. Some EO constituents could also act synergistically with some medicines, such as antibiotics, for increased efficacy [47]. All these studies and many others recommended the use of EOs in the management of skin damage, tissue repair/remodeling, control of homeostasis, or cancer treatment. It is well documented that the biological activities of the EOs depend on their chemical composition. However, although the Food and Drug Administration of USA (FDA) recognizes many EOs as Generally Recognized as Safe (GRAS), adverse reactions to specific compounds are commonly reported, manifesting as irritations and toxic effects [40]. For this reason, in the present study, we investigated the cytotoxic effects of oregano and tarragon EOs on external (A375) and internal (LNCaP and Caco2) human tumor cell lines for different potential uses.

The results indicated a slightly stronger cytotoxic activity of tarragon EO compared to oregano EO and similar to those of sage EO reported in a previous study [19]. Regarding the oregano EO (*O. vulgare*), other studies indicated that the cytotoxic effect on tumor cells in vitro is related to a higher concentration of carvacrol and thymol: 60.97% [41], 70.04% [39], and 74.8% [43]. Furthermore, Vimalanathan and Hudson [48] found that carvacrol showed high cytotoxicity alone. If we look at the volatile profile of the oregano and tarragon EOs obtained by us (Table 1), we notice that the two compounds were not found. This means that, in addition to carvacrol and thymol, other compounds could have a cytotoxic activity on tumor cells in vitro.

Studies by other authors have suggested that minor compounds can play a considerable role in cytotoxic activity, their antagonistic or synergistic effects being also possible [49]. However, the diversity of chemical composition requires testing the biologic activity for each EO produced.

A wide range of cell lines have been used to identify the cytotoxic or antiproliferative effects of oregano EO: human hepatocellular carcinoma (HepG2) and human embryonic kidney (HEK293) [43]; murine macrophage cells (RAW264.7) [42]; normal human keratinocyte (NCTC 2544) [41]; murine fibroblast (NIH-3T3); and human melanocyte (NGM) [40]. Alternately, the genotoxic effects of some EOs on HEL 12,469 human embryo lung cells were evaluated by Puškárová and collaborators [50] and the results revealed that none of these oils (from oregano, thyme, clove, lavender, clary sage, and arborvitae) induced significant DNA damage in vitro after 24 h.

There are fewer studies regarding the biological activity of tarragon EO, especially related to its cytotoxicity on cell lines. According to Sahakyan and collaborators [45], the sub-cytotoxic concentration of tarragon EO was 5.1 µg/mL on murine microglial BV-2 cell lines (WT and Acyl-CoA oxidase type 1 deficient mutant cells). Another study demonstrated that tarragon extracts have cytotoxic and antiproliferative effects on breast cancer cell lines MCF-7, T-47D, and MDA-MB-231 [51]. However, the cytotoxic effect on different types of cells is related as well to the chemical composition of the tarragon EO used. According to Ekiert and collaborators [14], the main components of the EO are estragole, otherwise known as methyl chavicol or p-allylanisole (40–85%), sabinene (approx. 35%), methyl eugenol (approx. 25%), and elemicin (up to 57%). By comparison to these values, or those obtained by other researchers [45,52], the ones observed in the present study are generally lower, especially in the case of estragole (chavicol methyl).

Availability and quality of the aromatic herbs are closely related to their potential in ensuring a reliable supply of plant-derived products such as EOs. Research on EOs from less known, rare, niche plant species, on those limited to certain geographical regions, or the ones which can be mainly sourced from the wild [53,54], although highly valuable, could potentially pose some issues for the supply chain. Given the fact that a reliable source of EO would be needed to establish a consistent or extended use of certain EOs, the cultivation becomes a requirement. Yet, devising cultivation technology for lesser-known species could take time. Moreover, the acceptability of the natural aroma that many such plant species have is another unknown, which could prove a limiting factor for the time being. Such aspects could delay their introduction or extending their use. Based on this, we argue that the best candidates remain, at this time, species that can be cultivated in regions where high demand of EOs is registered, such as Europe, and for which cultivation technology is well known and optimized at this current moment. In this regard, we strongly support the idea that culinary herbs are good candidates and should be researched further.

## 4. Materials and Methods

### 4.1. Herb Cultivation and Essential Oil Extraction

Herbs were cultivated in an experimental field located in the Agro-Botanical Garden of UASVM Cluj-Napoca, Romania, where the climate is temperate continental. Soil had alkaline reaction (pH 7.50), humus 3.30%, total nitrogen 0.155%, phosphorus 22 ppm, and potassium 185 ppm [19].

The experimental field was established in the year 2020 from seedlings obtained in a heated greenhouse. Seedlings were transferred to the field in the month of May. Tarragon seedlings (*A. dracunculus*) were planted at distances of 70 cm between rows and 30 cm between plants per row. Oregano seedlings (*O. vulgare*) were planted at 70 cm distance between rows and 25 cm between plants per row. Mechanical weed control was applied. No phytosanitary treatments were conducted.

In the year 2022, *herba* was harvested at the flowering stage (month of July) for EO extraction. The EO was obtained from fresh vegetal material by steam distillation using a 100 L capacity extractor (model E0141, Albrigi In Herba, Stallavena, Italy).

The EOs analyzed in this study has a guaranteed traceability, since all stages from cultivation to extraction were performed at USAMV Cluj-Napoca.

Plant voucher specimens were deposited at the Scientific Herbarium of the University of Agricultural Sciences and Veterinary Medicine of Cluj-Napoca, Romania; voucher numbers: CLA 30356 and CLA 30357.

### 4.2. GC-MS Volatile Profiling

Gas chromatographic analysis of the volatile compounds was performed using a GC-MS Shimadzu QP 2010 PLUS Mass Spectrometer coupled with Gas Chromatograph (Shimadzu equipped with an AOC-20i+s injector). A small amount from each EO sample was diluted in hexane and, from the obtained hexane–essential oil mixture, 1 µL was injected (split ratio of 1:50) in the GC-MS. The volatile organic compounds were separated on a ZB-5MS Plus capillary column (30 m × 0.25 mm, 0.25 µm film thickness, produced by Phenomenex), applying the following temperature program: starting from 60 °C (maintained for 1 min), the temperature was raised to 120 °C at a rate of 5 °C/min (maintained for 5 min); from 120 °C to 250 °C with 20 °C/min; and, at the end, to 300 °C at a rate of 30 °C/min and held for 2 min. The injector and the interface temperature were 250 °C, and the ion source temperature was set at 220 °C. A quadrupole mass spectrometer was used to detect the separated volatile organic compounds (VOC), in electron impact mode (EI, 70 eV), with an acquisition range (*m*/*z*) from 35 to 800 in scan mode, at an acquisition rate of 500 ms. The identification of volatile compounds was achieved based on their retention indices (RI) relative to n-alkanes (C6–C20) and by matching their mass spectra to the NIST (NIST 27, 147 libraries), WILEY library database (>90% match). The relative percentage of each VOC was estimated as a fraction of its integrated ion area from the total ion chromatogram (TIC) area (100%).

### 4.3. FT-IR Analysis

Fourier transform infrared spectroscopy (FT-IR) investigation of the EOs samples was performed using Fourier Transform Infrared Spectrometer (Jasco FT/IR 4100, Jasco, Tokyo, Japan). Measurements were conducted on KBr pellets with a drop of 2 μL EOs at the range of 4000–350 cm^−1^ and the scanning resolution 4.0 cm^−1^, with 256 readings per sample. Spectra were analyzed with Origin software (OriginLab, Northampton, MA, USA). The EO spectra were compared with standard IR spectra of major components found in the EOs from the National Institute of Standards and Technology (NIST, Gaithersburg, MD, USA) web-book data base.

### 4.4. Antibacterial Activity

#### 4.4.1. Preparation of Microbial Strains

The microorganisms tested were *Escherichia coli* ATCC 25922, *Salmonella enteritidis* ATCC 13076, *Staphylococcus aureus* ATCC 6538P, and *Listeria monocytogenes* ATCC 19114. Every strain was cultured for 24 h at 37 °C in a test tube with 10 mL of sterile nutritional broth (Oxoid Ltd., Basingstoke, Hampshire, UK). On a selective media, a loopful of inoculum was transferred: TBX agar for *E. coli*, XLD agar for *Salmonella enteritidis* (Oxoid Ltd., Basingstoke, Hampshire, UK), Baird–Parker agar base supplemented with Egg Yolk Tellurite Emulsion for *S. aureus*, and Palcam agar (Oxoid Ltd., Basingstoke, Hampshire, UK) for *Listeria monocytogenes* [55].

Bacterial morphology was confirmed by optical microscopy. After being grown on Mueller–Hinton agar (Oxoid Ltd., Basingstoke, Hampshire, UK) for 24 h at 37 °C, several colonies of standard cultures were transferred into sterile saline solution (8.5 g/L) and adjusted to the turbidity of the McFarland 0.5 standard (1.5 × 10^8^ CFU/mL) [56,57].

#### 4.4.2. Assessment of Minimum Inhibitory Concentration (MIC)

The resazurin microtiter-plate-based antibacterial test was used to calculate the MIC. The essential oils were dissolved into eight parts 50% ethanol and one part Tween 80 to create stock solutions [57]. A 96-well microtiter plate was used, and 100 µL of sterile nutrient broth and 100 µL of sample were placed into the first well. A total of 100 µL was transferred from well to well (on row) in order to perform serial 11-fold dilutions. From the last well of the row, 100 µL was discarded. To each well, 10 µL of inoculum (1.5 × 10^8^ CFU/mL) was added. A positive control of gentamicin (0.04 mg/mL in saline solution) was used. Eight parts 50% ethanol, one part Tween 80, and one part saline solution were combined to produce the mixture used as a negative control. Microplates were incubated for 22 h at 37 °C. To each well, 20 µL resazurin aqueous solution (0.2 mg/mL) was added. The microplates were incubated at 37 °C for 2 h. The concentration at which the blue hue did not turn pink was known as the minimum inhibitory concentration (MIC). For every sample, three replicates were performed [19].

#### 4.4.3. Assessment of the Minimum Bactericidal Concentration (MBC)

MBC was ascertained by plating a 10 μL aliquot from the final 4 wells that showed inhibition of bacterial growth in the MIC tests on Mueller–Hinton solid culture medium (Oxoid Ltd., Basingstoke, Hampshire, England). Afterwards, the plates were incubated for 24 h at 37 °C. Minimum bactericidal concentration was defined as the lowest concentration that stopped bacterial growth and left no colonies on the plate. For every plate, three distinct replicates were carried out [57,58].

### 4.5. Cytotoxic Effect on Cell Lines

The experiments were carried out on three human tumor cell lines: A375 (skin melanoma, ATCC^®^ CRL-1619), LNCaP (prostate carcinoma, ATCC^®^ CRL-1740), and Caco2 (colorectal adenocarcinoma, ATCC^®^ HTB-37). A375 cell line was cultivated in Dulbecco’s modified Eagle medium (DMEM) with 2 mM L-glutamine and 10% fetal bovine serum (FBS). For LNCaP cells, the complete growth medium was RPMI-1640 Medium with 2 mM L-glutamine and 10% FBS, while, for Caco2 cells, Eagle’s minimum essential medium (MEM) with 2 mM L-glutamine, 1 mM sodium pyruvate, 1% (*v/v*) NEAA, and 10% (*v/v*) FBS was used. All these cultures were performed in media without antibiotics, in an atmosphere with 5% CO_2_ and 95% humidity at 37 °C (Memmert, INCO2).

At 80-90% confluence, cell lines were detached using 0.25% (*w/v*) trypsin and 0.53 mM EDTA solution and the cells were seeded on 96-well plates at a density of 1 × 104 cells per well in 200 µL culture medium. After 24 h, the cells were treated with different concentrations: 0.001, 0.002, 0.004, 0.008, 0.016, 0.032, and 0.064% (*v/v*) for each EO (oregano and tarragon), then incubated for the next 24 h. Culture medium with cells was used as the control. Tween 20 was used as a solvent (10%) for stock solutions and its cytotoxic activity was tested as well at the maximum concentration of 0.064%. The stock solutions were prepared on the day of testing. The cytotoxic effect after 24 h exposure was examined with contrast phase microscopy (Olympus IX51, Olympus, Tokyo, Japan). The cytotoxicity was evaluated by using 3-(4,5-dimethyl-2-thiazolyl)-2,5-diphenyl-2H-terazolium bromide (MTT) assay. The cells were washed with phosphate-buffered saline (PBS), then 150 µL/well MTT solution (5 mg/mL) was added and incubated for 1 h at 37 °C. After incubation, the formazan crystals were dissolved in 150 µL/well dimethyl sulfoxide (DMSO). The absorbance was measured at 550 nm and 630 nm with an HT BioTek Synergy microplate reader (BioTek Instruments, Venusky, VT, USA). Results were expressed as percentages of control cells. The experiments were performed in triplicate.

### 4.6. Statistical Analysis

For the biologic activity, the data were reported as average mean ± standard deviation (SD) for triplicate determinations.

In the case of microbiological activity, the ANOVA analysis of variance was used to compare the average mean values, using SPSS 19.0 statistical analysis (IBM, New York, NY, USA) and Tukey’s honestly significant differences (HSD) test with a confidence interval of 95% or 99%. A *p*-value below 0.05 was considered statistically significant.

For the cytotoxic effect, IC_50_ values were calculated in order to detect differences by using GraphPad Prism (Boston, MA, USA).

## 5. Conclusions

This research reports the chemical profile, composition, and biologic activities of two EOs obtained from organic crops of oregano and tarragon located in Cluj, Central Transylvania, Romania. In response to the four objectives of this study, it was found that (1) the oregano EO presented 22 compounds and the volatile profile was dominated by sesquiterpenes, while the major constituent was β-caryophyllene (26.89%); the tarragon EO presented 20 compounds and the volatile profile was dominated by phenylpropanoids, while the major constituent was eugenol (29.19%); (2) FT-IR spectrum of oregano EO presented a characteristic strong band between 2800 and 3000 cm^−1^, while FT-IR spectrum of tarragon EO displayed characteristic sharp peaks between 1000 and 1600 cm^−1^ due to phenylpropanoids, which accounted for >50 of EO; (3) oregano EO compared to tarragon EO had a stronger antibacterial effect against both Gram-positive (*S. aureus* and *L. monocytogenes*) as well as Gram-negative (*E. coli* and *S. enteritidis*) bacterial strains; (4) tarragon EO compared to oregano EO had a slightly stronger cytotoxic effect on three types of cancer cell lines (skin melanoma, prostate carcinoma, and colorectal adenocarcinoma).

The preliminary results suggest a potential for applications at least for food pathogen control, as well as for some external and possibly internal uses.

There are several arguments in favor of culinary plants (such as oregano and tarragon) compared to other aromatic herbs to be strongly considered for EO extraction, study, and eventually diversified applications. These could represent criteria as well when considering the potential of other EO-producing plants. Among the main practical advantages is the fact that many popular culinary herbs such as the ones from this study can be cultivated in regions where high demand for EOs exists, cultivation technology is well known, and their aroma is acceptable to most people.

Careful attention has to be given to rigorous determination of cultivars, since previous studies have shown a great variation in the chemotypes, at least for oregano, which was studied to a higher extent than tarragon to date. For a transparent and safe customer experience, traceability should become a mainstream requirement for EOs.

## Figures and Tables

**Table 1:** Volatile profile of oregano and tarragon essential oils (expressed as Area%).

Class	Compound	Oregano	Tarragon
bicyclic monoterpene	Sabinene	6.32	9.44
acyclic monoterpene	*trans*-β-Ocimene	2.85	2.21
acyclic monoterpene	Linalool	1.85	0.38
acyclic monoterpenoids	(4E,6Z)-allo-Ocimene	5.18	2.40
acyclic monoterpenoids	Linalool acetate	3.26	-
acyclic monoterpenoid	4-Terpineol	2.35	2.72
acyclic monoterpenoid	α-Terpineol	0.4	1.13
monoterpene	β-Citronellol	-	4.74
monoterpene	Geranyl acetate	-	3.18
monoterpene	D-Carvone	-	1.11
phenylpropanoid	Methyl eugenol	-	29.19
phenylpropanoid	Isoelemicin	-	16.33
phenylpropanoid	Elemicin	-	11.43
phenylpropanoid	Methylisoeugenol	-	2.85
phenylpropanoid	p-Allyl Anisole/Chavicol methyl ester	-	1.13
sesquiterpene	α-Bergamotene	-	1.17
sesquiterpene	β-Caryophyllene	26.89	2.09
sesquiterpene	α Bisabolene	1.31	0.39
sesquiterpene	β-Cubebene	6.95	-
sesquiterpene	β-Bourbonene	5.40	-
sesquiterpene	Germacrene	4.05	-
sesquiterpene	α-Farnesene	2.95	-
sesquiterpene	γ-Muurolene	2.86	-
sesquiterpene	δ Cadinene	2.57	-
sesquiterpene	γ-Cadinene	1.63	-
sesquiterpene	γ-Elemene	1.51	-
sesquiterpene	(E,E)-α-Farnesene	1.48	-
monocyclic sesquiterpene	α-Caryophyllene	2.74	-
sesquiterpene alcohol	-Spathulenol	2.39	0.82
sesquiterpenoid oxide	Caryophyllene oxide	2.42	0.44
sesquiterpenoid	α-Cadinol	1.71	0.20

**Table 2:** Antibacterial activity of oregano and tarragon essential oils against Gram-negative and Gram-positive bacteria, assessed by broth microdilution testing.

Samples	*Escherichia coli* ATCC 25922		*Salmonella enteritidis* ATCC 13076		*Staphylococcus aureus* ATCC 6538P		*Listeria monocytogenes* ATCC 19114	
	MIC (μL/mL)	MBC (μL/mL)	MIC (μL/mL)	MBC (μL/mL)	MIC (μL/mL)	MBC (μL/mL)	MIC (μL/mL)	MBC (μL/mL)
Oregano	0.27 ± 0.00 ^b^	1.17 ± 0.00 ^b^	0.095 ± 0.73 ^b^	0.27 ± 0.00 ^b^	0.42 ± 0.73 ^b^	0.56 ± 0.00 ^b^	0.87 ± 0.73 ^b^	2.45 ± 0.00 ^b^
Tarragon	2.45 ± 0.00 ^a^	10.80 ± 0.00 ^a^	1.17 ± 0.00 ^a^	5.14 ± 0.00 ^a^	5.14 ± 0.00 ^a^	10.80 ± 0.00 ^a^	2.45 ± 0.00 ^a^	10.80 ± 0.00 ^a^
Gentamicin (μg/mL)	0.24 ± 0.00	0.24 ± 0.00	0.50 ± 0.73	0.50 ± 0.73	0.05 ± 0.73	0.05 ± 0.73	0.50 ± 0.00	0.50 ± 0.00

Note: mean values of three replicates ± SD. Values followed by different letters (^a,b^) within a column express significant differences (*p* < 0.05) based on Tukey’s honestly significant differences (HSD) test, where confidence intervals of 95% or 99% were considered; minimum bactericidal concentration (MBC), minimum inhibitory concentration (MIC).

## Data Availability

Data from this study are available in the text of the paper.
